# Successful Latissimus Dorsi Flap Reconstruction Following Intraoperative Division of the Thoracodorsal Pedicle During Breast Cancer Surgery: A Case Report

**DOI:** 10.7759/cureus.110885

**Published:** 2026-06-15

**Authors:** Aditya S S N Kalyan Kondeti, Balasubramanian Venkitaraman, Suhaildeen Kajamohideen

**Affiliations:** 1 Surgical Oncology, Sri Ramachandra Institute of Higher Education and Research, Chennai, IND

**Keywords:** breast reconstruction, indocyanine green angiography, latissimus dorsi flap, segmental perforators, thoracodorsal artery

## Abstract

The latissimus dorsi (LD) musculocutaneous flap remains a reliable option for breast and chest wall reconstruction because of its consistent anatomy and dependable vascularity. The flap is traditionally considered dependent on the thoracodorsal vascular pedicle, and division of this pedicle is often regarded as a contraindication to its use. We report the case of a 53-year-old woman with locally advanced right breast carcinoma who underwent modified radical mastectomy following neoadjuvant systemic therapy. During axillary dissection, the thoracodorsal vessels were intentionally ligated and divided because of dense adherence to metastatic lymph nodes, whereas the angular branch and multiple intercostal and lumbar perforators were preserved. Given the extensive postmastectomy defect, reconstruction using an LD flap was considered. Intraoperative indocyanine green (ICG) angiography demonstrated satisfactory flap perfusion despite division of the dominant pedicle, allowing successful flap inset. The postoperative course was uneventful, with only minimal marginal flap necrosis that resolved with conservative management and without the need for additional surgical intervention. This case demonstrates that thoracodorsal pedicle division does not necessarily preclude successful LD flap reconstruction when collateral vascular pathways are preserved. Intraoperative ICG angiography provides valuable real-time assessment of flap perfusion and may facilitate safe reconstructive decision-making in challenging oncologic situations.

## Introduction

The latissimus dorsi (LD) musculocutaneous flap remains one of the most versatile and reliable options for breast and chest wall reconstruction owing to its predictable anatomy and robust vascular supply. The thoracodorsal artery, a branch of the subscapular artery, constitutes the dominant vascular pedicle of the LD muscle. Distally, it gives rise to the angular branch, which courses toward the inferior angle of the scapula and may contribute significantly to collateral perfusion.

According to the Mathes and Nahai classification, the LD muscle is a Type V flap supplied by a dominant thoracodorsal vascular pedicle and secondary segmental perforators arising from the intercostal and lumbar vessels [1]. Traditionally, the thoracodorsal artery has been regarded as the principal blood supply to the LD flap, and injury to or intentional sacrifice of this pedicle during oncologic surgery is often considered a contraindication to flap utilization. Nevertheless, anatomical and clinical studies have demonstrated that collateral circulation through the angular branch and segmental perforators may be sufficient to maintain flap viability even after division of the dominant pedicle [1-3].

Indocyanine green (ICG) angiography has emerged as a valuable adjunct in reconstructive surgery by providing real-time assessment of tissue perfusion and assisting intraoperative decision-making when vascularity is uncertain [4,5]. We report a case of successful LD flap reconstruction following intentional intraoperative division of the thoracodorsal pedicle during modified radical mastectomy, with flap viability confirmed using ICG angiography.

## Case presentation

**Figure 1:**
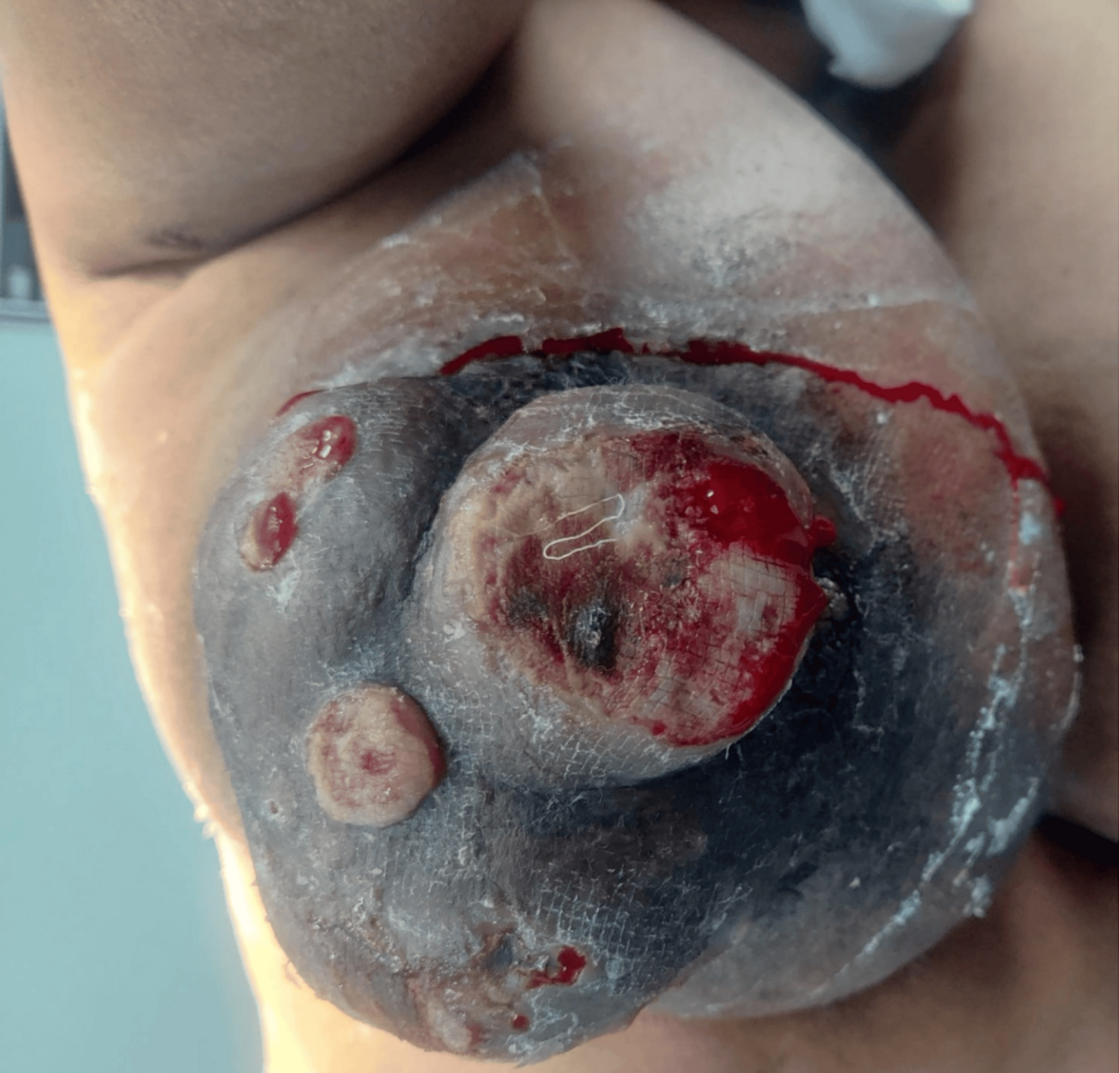
Preoperative clinical photograph demonstrating locally advanced carcinoma of the right breast

A 53-year-old female presented with a locally advanced carcinoma of the right breast, clinically staged as cT4bN2M0 (Figure 1). PET-CT demonstrated no evidence of distant metastatic disease.

The patient initially received neoadjuvant chemotherapy consisting of doxorubicin (Adriamycin) and cyclophosphamide. Owing to disease progression after two cycles, treatment was modified to docetaxel and trastuzumab for three additional cycles, after which definitive surgical management was planned.

**Figure 2:**
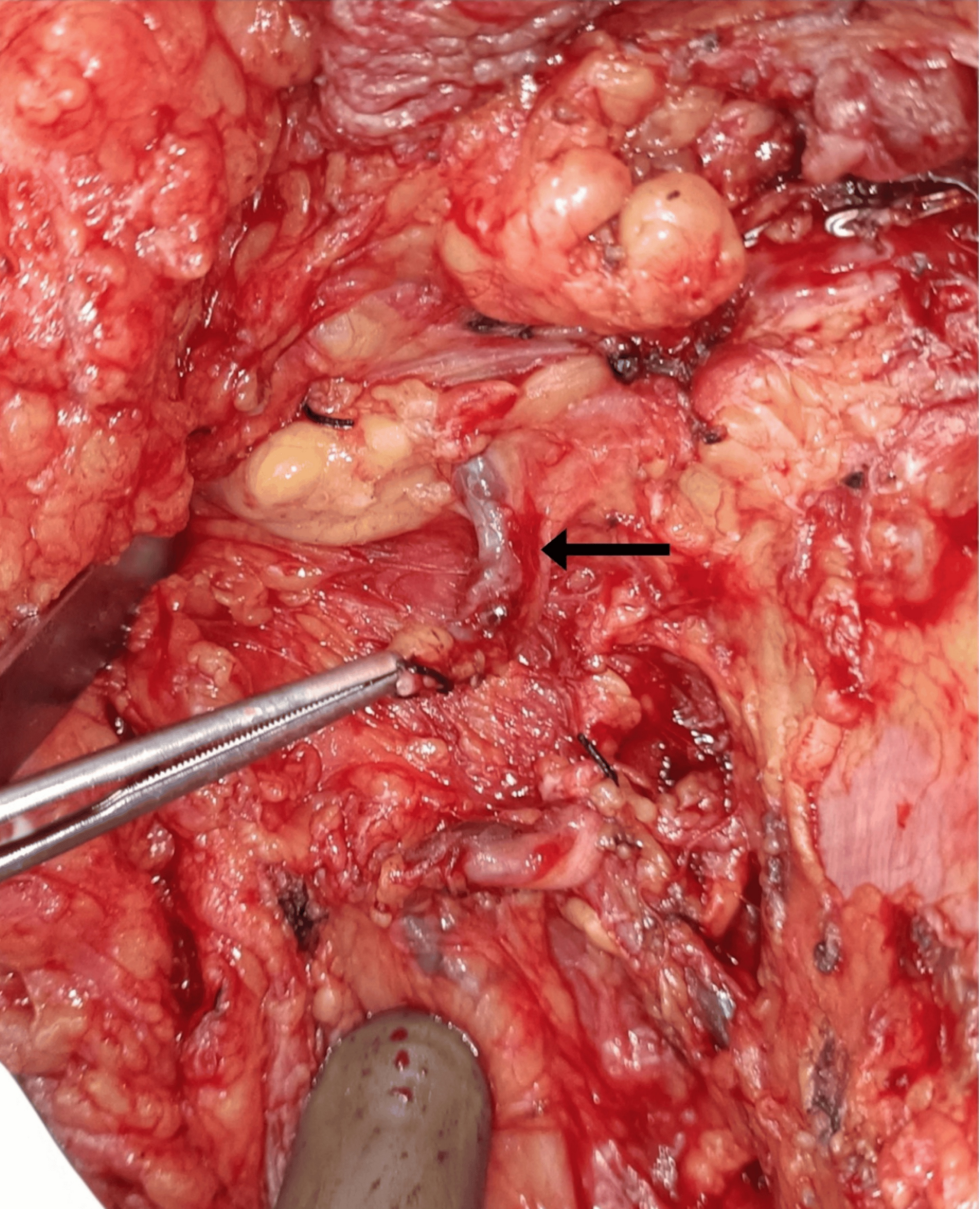
Intraoperative photograph demonstrating ligation and division of the thoracodorsal vascular pedicle during axillary dissection

**Figure 3:**
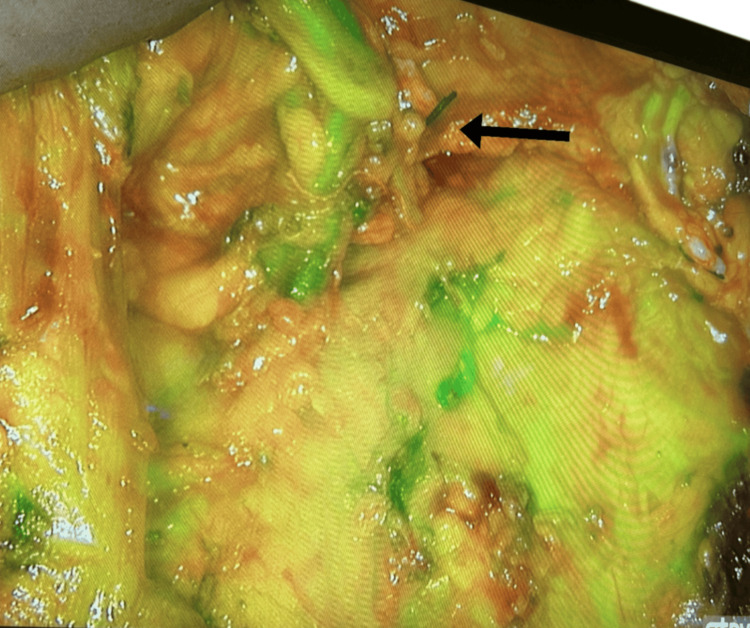
Intraoperative overlay view showing the divided thoracodorsal pedicle (ICG) ICG, indocyanine green

The patient subsequently underwent a right modified radical mastectomy. During axillary dissection, multiple metastatic lymph nodes were found to be densely adherent to the thoracodorsal vascular pedicle. To achieve adequate oncologic clearance, the thoracodorsal artery and vein were intentionally ligated and divided (Figures 2, 3).

**Figure 4:**
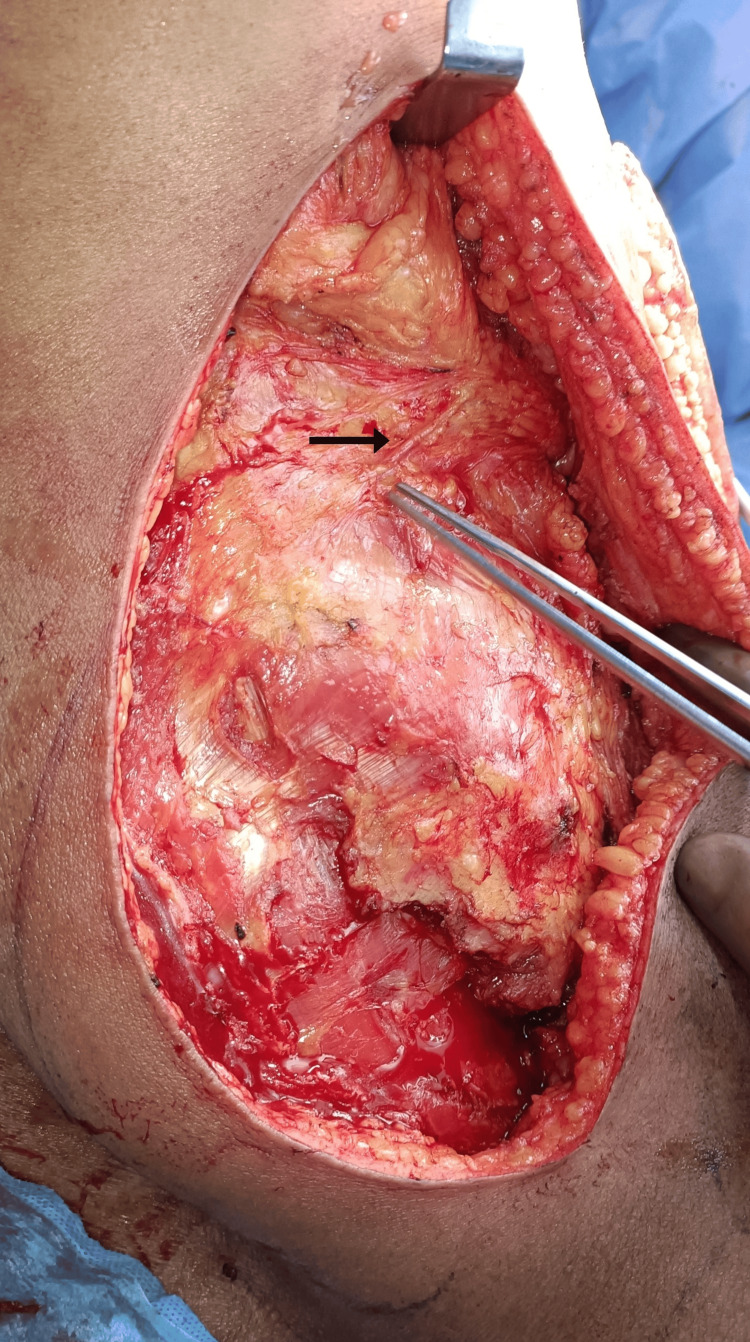
Preserved lumbar and intercostal perforators identified during flap elevation

The thoracodorsal vessels were divided distal to the origin of the angular branch. Given the anticipated soft-tissue defect following mastectomy, reconstruction using a pedicled LD flap was considered. During flap harvest, multiple posterior intercostal and lumbar perforators were preserved (Figure 4). A skin paddle measuring approximately 20 × 10 cm was harvested and rotated approximately 80° into the mastectomy defect without significant tension. Preservation of the collateral vascular pathways did not substantially restrict flap reach or inset, allowing satisfactory coverage of the postmastectomy defect.

**Figure 5:**
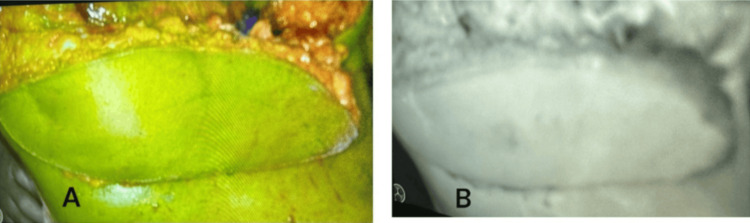
Intraoperative ICG angiography demonstrating satisfactory perfusion of the LD flap despite division of the dominant thoracodorsal pedicle (A) Overlay mode. (B) Monochromatic mode. ICG, indocyanine green; LD, latissimus dorsi

Because the dominant vascular pedicle had been sacrificed, intraoperative assessment of flap viability was performed using ICG angiography. After intravenous injection of 2 mL (5 mg) of Aurogreen, fluorescence became evident approximately 20-30 seconds after ICG injection, with progressive homogeneous enhancement of the central flap and no major areas of persistent hypoperfusion, with slightly delayed but satisfactory perfusion at the medial margin (Figure 5). Based on these findings, the flap was deemed viable, and the inset was completed.

**Figure 6:**
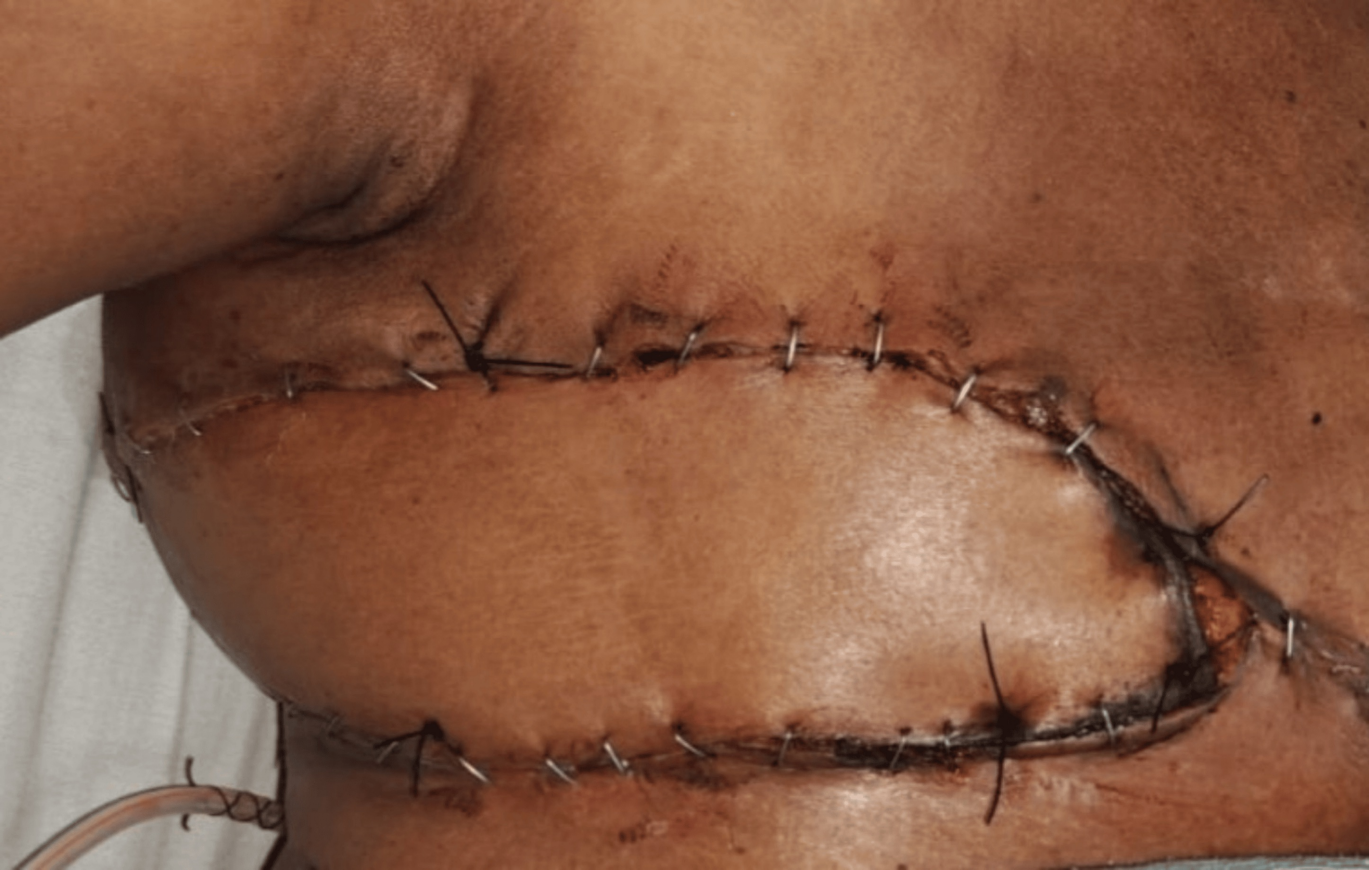
Postoperative appearance and early follow-up demonstrating satisfactory flap viability and wound healing

The postoperative course was uneventful (Figure 6). The patient was discharged on postoperative day 4 with surgical drains in situ.

At the two-week follow-up, the flap remained viable with satisfactory wound healing. A small area of marginal necrosis was noted along the medial edge of the flap. This was managed conservatively with local wound care and healed without the need for further surgical intervention. No evidence of flap loss, infection, or major wound-related complications was observed during subsequent follow-up.

## Discussion

The LD flap continues to be a cornerstone of reconstructive surgery because of its dependable anatomy, versatility, and favorable complication profile. Although traditionally considered dependent on the thoracodorsal artery, several anatomical studies have demonstrated the existence of substantial collateral vascular networks capable of supporting flap viability following compromise of the dominant pedicle [2,6].

These collateral pathways include perforators arising from the posterior intercostal, subcostal, and lumbar arteries, as well as the angular branch associated with the thoracodorsal system. Feng et al. described the vascular anatomy of the pedicled LD flap and emphasized the contribution of these secondary vascular territories in maintaining tissue perfusion [7]. Similarly, Merkkola-von Schantz et al. highlighted the versatility of the LD flap and its capacity to tolerate varying degrees of vascular compromise [8].

Clinical reports have documented successful LD flap survival despite thoracodorsal vessel division. Particular attention has been directed toward identifying and preserving the angular branch during axillary dissection. The angular branch represents an important collateral vascular pathway and may contribute significantly to flap perfusion following sacrifice of the thoracodorsal pedicle. Preservation of this branch, together with intercostal and lumbar perforators, likely contributed to the favorable outcome observed in the present case.

Hartmann et al. reported successful breast reconstruction using a pedicled LD flap in the absence of an intact thoracodorsal pedicle, suggesting that collateral circulation may provide adequate perfusion under selected circumstances [1]. Additional reports have described flap survival based on intercostal or lumbar perforator systems after sacrifice of the dominant pedicle [2,3].

ICG angiography has become increasingly popular in reconstructive surgery as a means of objectively assessing tissue perfusion. Unlike conventional clinical evaluation alone, ICG angiography allows visualization of vascular inflow and tissue perfusion in real time. Previous studies have demonstrated its utility in reducing rates of partial flap necrosis and facilitating intraoperative decision-making [4,5].

In the present case, ICG angiography provided objective confirmation of adequate flap perfusion despite deliberate thoracodorsal pedicle division. This information allowed reconstruction to proceed confidently and avoided abandonment of a potentially viable flap. The favorable postoperative outcome further supports the importance of preserving collateral vascular pathways whenever sacrifice of the dominant pedicle is unavoidable.

To our knowledge, reports describing immediate LD flap reconstruction following intentional thoracodorsal pedicle division during oncologic surgery remain limited. This case highlights the practical value of understanding collateral vascular anatomy and demonstrates the usefulness of ICG angiography in confirming flap viability before definitive reconstruction.

## Conclusions

This case suggests that successful LD flap reconstruction may be feasible in selected patients despite thoracodorsal pedicle division when collateral circulation is preserved and adequate perfusion is confirmed intraoperatively. Preservation of the angular branch and segmental perforators may provide sufficient collateral perfusion to maintain flap viability. Intraoperative ICG angiography offers an objective method of assessing vascularity and can facilitate safe reconstructive decision-making when the dominant vascular pedicle has been compromised. Awareness of these alternative vascular pathways may expand reconstructive options in complex oncologic cases. Further clinical experience is required before broader conclusions can be drawn.
